# Prevalence and Antimicrobial Resistance of Enteropathogenic Bacteria in Yellow-Legged Gulls (*Larus michahellis*) in Southern Italy

**DOI:** 10.3390/ani11020275

**Published:** 2021-01-22

**Authors:** Tamara Pasqualina Russo, Antonino Pace, Lorena Varriale, Luca Borrelli, Antonio Gargiulo, Marina Pompameo, Alessandro Fioretti, Ludovico Dipineto

**Affiliations:** 1 Department of Veterinary Medicine and Animal Productions, Università degli Studi di Napoli Federico II, via Delpino 1, 80137 Naples, Italy; antonino.pace@szn.it (A.P.); lorena.varriale@unina.it (L.V.); luca.borrelli@unina.it (L.B.); angargiulo@libero.it (A.G.); fioretti@unina.it (A.F.); ludovico.dipineto@unina.it (L.D.); 2 Marine Turtle Research Centre, Stazione Zoologica Anton Dohrn, via Nuovo Macello 16, 80055 Portici, Italy; 3 Veterinary Hospital, ASL Napoli 1 Centro, via M. Rocco di Torrepadula 13, 80145 Naples, Italy; marina.pompameo@aslnapoli1centro.it

**Keywords:** yellow-legged gull, *Salmonella*, *Campylobacter*, Shiga toxin-producing *E. coli*, zoonosis, antimicrobial resistance, public health

## Abstract

**Simple Summary:**

Several Mediterranean countries have suffered from a significant increase in the populations of gulls. These birds are agile fliers and are able to adapt easily to different habitats and to profit from food discarded by humans. In addition, gulls may be considered by some as pests, given their interactions with human activities, and have been described as carriers, but actually function as environmental sentinels of enteropathogenic bacteria and antibiotic-resistant strains. Therefore, the aim of this study was to examine the role of gulls as vectors of zoonotic agents of high importance for human health and as potential reservoirs of antimicrobial-resistant strains.

**Abstract:**

Wild birds may host and spread pathogens, integrating the epidemiology of infectious diseases. Particularly, *Larus* spp. have been described as responsible for the spread of many enteric diseases, primarily because of their large populations at landfill sites. The aim of this study was to examine the role of yellow-legged gulls as a source of enteropathogenic bacteria such as *Campylobacter* spp., *Salmonella* spp., Shiga toxin-producing *Escherichia coli* and *Yersinia* spp., with particular attention to antibiotic-resistant strains. Enteropathogenic bacteria were isolated from 93/225 yellow-legged gulls examined from April to July, during a four-year period (2016–2019). Specifically, *Campylobacter* spp. was isolated from 60/225 samples (26.7%), and identified as *C. coli* (36/60) and as *C. jejuni* (24/60). *Salmonella* spp. was isolated from 3/225 samples (1.3%), and identified as *Salmonella arizonae*. Shiga toxin-producing *E. coli* were isolated from 30/225 samples (13.3%) samples, and serotyped as *E. coli* O128 (12/30) O26 (9/30), O157 (6/30) and O11 (3/30); *Yersinia* spp. was never detected. Isolated strains exhibited multidrug resistance, including vitally important antibiotics for human medicine (i.e., fluoroquinolones, tetracyclines). Our study emphasizes the importance of yellow-legged gulls as potential reservoirs of pathogenic and resistant strains and their involvement in the dissemination of these bacteria across different environments, with resulting public health concerns.

## 1. Introduction

The yellow-legged gull (*Larus michahellis*) (Laridae) inhabits different kinds of habitats, from seashores, lakes, and rivers to farmlands and urban surroundings. Indeed, the yellow-legged gull is considered an opportunistic species, given its adaptability to different environments. The Italian yellow-legged gull population, as in most Mediterranean countries, experienced an intense increase (58–125%) during the second half of the 1900s, reaching 45–60 thousand breeding pairs in 2000, from 24–27 thousand pairs estimated in 1983 [1]. This growth was mainly caused by the mitigation of the negative human impact on gull colonies on one side, and by the increase in trophic sources of human origins (e.g., discarded material or waste) on the other [1]. As a result, the yellow-legged gull has become a troublesome species all over the Mediterranean area, drawing adverse considerations regarding its interactions with humans and other animals, which can be negatively affected by its aggressive behaviour [2]. Gulls are classified as generalist foragers, given their wide range of prey, but they are also considered opportunists, because they can also feed on waste of human origin and carrion [3]. The combination of different habitats and feeding habits makes gulls vulnerable of encountering a wide spectrum of microorganisms [3]. Therefore, gulls have been considered valid sentinel species, especially to explore the influence of urbanization on microbial communities [4]. Indeed, gulls act as potential reservoirs of pathogenic and antibiotic-resistant bacteria, and might spread these strains across the different environments they inhabit [4,5]. The dramatic growth of the urban gull populations raises important health concerns, especially considering that the routes of acquisition and dissemination have not been elucidated, thus impeding the development of appropriate control measures [6]. Gulls might host several microorganisms, including enteropathogenic bacteria such as *Salmonella*, *Campylobacter* and Shiga toxin-producing *E. coli* (STEC). These bacterial agents have been recognized as mainly responsible for human enteric diseases, and have been included by the World Health Organization among those with the evident ability to cause infection in humans following transmission from non-human sources, which should be identified [6,7,8,9]. Indeed, in 2017, *Campylobacter* spp. was reported as the most frequent bacterial cause of human gastroenteritis in Europe since 2005, followed by *Salmonella* spp., *Yersinia* spp., and STEC. Similarly, in 2018, *Salmonella* spp. was reported as the most common food-borne pathogen and the second most frequent zoonotic agent in Europe [8].

Another reason of concern is the overuse of disinfectants and antimicrobials, which have induced a selective pressure on microorganisms during the last decades, acquiring crucial importance when considering bacterial resistance to antibiotics commonly used in human medicine [9]. In this context, antibiotic-resistance might be considered a zoonosis, because resistant strains are transferred among wildlife, domestic animals, and humans, resulting in new reservoirs in the environment and concurring to the amplification and dissemination of antimicrobial resistance [10].

Several studies, in different European countries and in the Mediterranean basin, have reported enteropathogenic bacteria in gulls, including resistant strains, and suggested the adoption of these animals as indicators of antibiotic resistance in the environment [10,11,12,13,14,15]. Given the paucity of information on the subject in southern Italy, the aim of this study was to examine the role of gulls as vectors of zoonotic agents of high importance for human health and as potential reservoirs of antibiotic-resistant strains. Specifically, this study focused on the yellow-legged gull population of the city of Naples, in order to estimate the prevalence of *Salmonella* spp., *Campylobacter* spp., STEC, and *Yersinia* spp., concurrently evaluating the antibiotic resistance of the isolated bacterial strains.

## 2. Materials and Methods

### 2.1. Sampling

From the beginning of April to the end of July, in the four-year period 2016–2019, a total of 225 yellow-legged gulls from the Campania region and recovered at the Wildlife Rescue and Rehabilitation Centre of the University of Naples Federico II were examined. Birds were sampled at their arrival at the centre and a cloacal swab was collected from each animal, using sterile cotton-tipped swabs. Swab samples were placed into 800 µL phosphate-buffered saline (PBS) and transported at 4 °C to the laboratory of the Department of Veterinary Medicine and Animal Productions of the University of Naples Federico II. Sampling procedures are part of the standard clinical evaluation and routine diagnostic testing of recovered wild birds, in accordance with the current legislation (Directive 2010/63/EU).

### 2.2. Bacterial Isolation

Samples were processed in order to isolate *Campylobacter* spp., *Salmonella* spp., STEC, and *Yersinia* spp., following the methods described by the ISO procedures and Söderlund et al. [16,17,18], with minor modifications (detailed below).

*Campylobacter* spp.: 100 µL of PBS were transferred into 10 mL of *Campylobacter* selective enrichment broth (Oxoid, UK) and incubated in a microaerophilic atmosphere (8–9% oxygen level and <8% carbon dioxide level, as provided by CampyGen, Oxoid) at 42 °C for 48 h. Subsequently, each sample was plated onto *Campylobacter* blood-free selective agar (CCDA; Oxoid, UK) and incubated in a microaerophilic atmosphere at 42 °C for 48 h. After incubation, the plates were examined for characteristic *Campylobacter* colonies, which were sub-cultured on sheep blood agar (Oxoid, UK) at 42 °C for 24 h. Colonies, after Gram staining, were examined by phase contrast microscopy, and those exhibiting curved or spiral motile rods were submitted to a multiplex polymerase chain reaction (PCR) for species confirmation, as described by Dipineto et al., 2017 [19].

*Salmonella* spp.: 100 µL of PBS were transferred into 10 mL of Buffered Peptone Water (Oxoid, UK) and incubated at 42 °C for 24 h. Subsequently, an aliquot of each samples was inoculated onto Rappaport-Vassiliadis broth (Oxoid, UK) and incubated at 42 °C for 18 h. After incubation, samples were streaked onto Xylose Lysine Deoxycholate agar (Oxoid, UK) and Brilliant Green Agar (Oxoid, UK), and incubated at 37 °C for 24 h. Suspected colonies were sub-cultured on Rambach agar (Merck) and in Triple Sugar Iron agar (Oxoid, UK) at 37 °C for 24 h and then examined for characteristic *Salmonella* colonies. All *Salmonella* isolated were identified using the miniaturized biochemical system API20E (Biomerieux, Italy).

*Yersinia* spp.: 100 µL of transport media were pre-enriched for 21 days into 10 mL of PBS at 4 °C. Every seven days, the samples were plated onto *Yersinia* Selective Agar–CIN Medium (Oxoid, UK), incubated at 30 °C for 24–48 h, and examined for characteristic *Yersinia* colonies.

Shiga toxin-producing *E. coli*: 100 µL of PBS were transferred into 10 mL of modified Tryptone Soy Broth (Oxoid, UK), with Novobiocin added (Oxoid, UK). Samples were incubated at 37 °C for 12–18 h and then plated onto Sorbitol MacConkey agar (Oxoid, UK) with added cefixime–tellurite (Oxoid, UK) and onto Sorbitol MacConkey agar with BCIG (Oxoid, UK), both incubated at 37 °C for 18–24 h. Colourless colonies on both media were presumptively identified as *E. coli* O157, whereas coloured colonies on both media were presumptively identified as other *E. coli.* Both colourless and coloured colonies grown on the selective media were subcultured on Nutrient agar at 37 °C for 18–24 h and were sero-grouped on the basis of their O antigen, using anti-coli polyspecific (I, II, III) and monospecific sera (Sifin, Germany), as well as an *E. coli* O157 latex test kit (Oxoid, UK). *E. coli* results positive to rapid serum agglutination were subcultured on washed sheep blood plates at 37 °C for 18–24 h, and then submitted to multiplex PCR, in order to determine the presence of Shiga toxin (stx1 and stx2) and *E. coli* attaching and effacing (eae) genes. DNA extraction and PCR amplification were performed as previously described [20,21], and PCR products were analysed in a 1.5% agarose gel stained with ethidium bromide (Gibco-BRL, Milan, Italy). A strain of *E. coli* O157 (ATCC 43894) and working solution without DNA were used as positive and negative controls, respectively.

### 2.3. Antimicrobial Susceptibility Testing

Isolated strains were subjected to antimicrobial susceptibility testing, using the disk diffusion technique, in accordance with the criteria established by the European Committee on Antimicrobial Susceptibility Testing [22] and the Clinical and Laboratory Standards Institute (CLSI) [23,24]. *Campylobacter* isolates were streaked onto Mueller-Hinton agar with 5% defibrinated sheep blood added (Oxoid, UK), and incubated, with antimicrobial disks, in microaerophilic conditions at 37 °C for 48 h. *Campylobacter* strains were tested with the following antimicrobial agents: azythromicin (AZM, 15 μg), chloramphenicol (CHL, 30 μg), ciprofloxacin (CIP, 5 μg), doxycycline (DO, 30 μg), enrofloxacin (ENR, 5 μg), erythromycin (E, 15 μg), gentamicin (CN, 10 μg), nalidixic acid (NA, 30 μg) and tetracycline (TE, 30 μg). Similarly, *Salmonella* and STEC isolates were streaked onto Mueller-Hinton agar (Oxoid, UK) and then incubated, with antimicrobial disks, at 37 °C for 24 h. The tested antimicrobial agents were: amoxicillin (AMO, 30 μg), amoxicillin–clavulanate (AMC, 20  +  10 μg), ampicillin (AMP, 10 μg), apramycin (APR, 40 μg), ceftazidime (CAZ, 30 μg), chloramphenicol (CHL, 30 μg), ciprofloxacin (CIP, 5 μg), colistin sulphate (CS, 10 μg), doxycycline (DO, 30 μg), enrofloxacin (ENR, 5 μg), gentamicin (CN, 10 μg), nalidixic acid (NA, 30 μg) streptomycin (S, 10 μg), sulphonamides compound (S3, 300 μg), sulphamethoxazole–trimethoprim (SXT, 1.25  +  23.75 μg), and tetracycline (TE, 30 μg). The antibiotics for susceptibility testing were chosen among the most commonly used molecules in human and animal medicine, with available and standardized breakpoints. For all strains the inhibition zones were measured and classified as susceptible, intermediate and resistant, in accordance with the CLSI document [25]. The presence of Extended Spectrum Beta-Lactamase (ESBL)-producing bacteria was evaluated, submitting all strains to the ETEST^®^ ESBL (ESBL CT/CTL 16/1; bioMérieux) and to the combination disk diffusion test, using cefpodoxime (CPD, 10 μg; Oxoid) and cefpodoxime/clavulanic acid (CD, 10/1 μg; Oxoid).

## 3. Results

The bacteriological survey revealed that 93/225 gulls (41.3%; 95% confidence interval (CI) = 34.9–48.1%) were positive for enteropathogenic bacteria, with no co-infection recorded (Table 1). Samples processed for *Yersinia* spp. were consistently negative.

*Campylobacter* spp. were isolated from 60/225 (26.7%; 95% CI = 21.4–32.8%) samples. Among these, as confirmed by multiplex PCR, 36/60 (60%) were identified as *C. coli* and 24/60 (40.0%) were identified as *C. jejuni*. All *Campylobacter* tested were susceptible to chloramphenicol and gentamicin (Table 2). The main antimicrobial resistances detected for *C. jejuni* and *C. coli*, were to tetracycline (62.5% and 52.8%, respectively), ciprofloxacin (37.5% and 33.3%, respectively) and nalidixic acid (37.5% and 27.7%, respectively).

When considering resistance to multiple antibiotics, among the 36 *C. coli* strains, nine (25.0%) were simultaneously resistant to tetracycline and ciprofloxacin; three (8.3%) were simultaneously resistant to tetracycline, ciprofloxacin, and erythromycin; and three (8.3%) were also resistant to azythromicin and nalidixic acid, in addition to the previous antibiotics. Among the 24 *C. jejuni* strains, six (25.0%) were simultaneously resistant to azythromicin, ciprofloxacin and tetracycline; four (33.3%) were simultaneously resistant to azythromicin, ciprofloxacin, tetracycline, and erythromycin; and two (8.3%) were also resistant to nalidixic acid, in addition to the previous antibiotics.

*Salmonella* spp. were isolated from 3/225 samples (1.3%; 95% CI 0.5–3.8%), and all strains were identified as *Salmonella arizonae*. One strain was susceptible to all tested antibiotics, whereas the other two strains (66.6%) were resistant only to sulphonamides compound.

*E. coli* were isolated from 189/225 samples (84.0%; 95% CI 78.7–88.2%), but Shiga toxin-producing *E. coli* were recovered only in 30/225 gulls (13.3%; 95% CI 9.5–18.3%) and classified according to their O antigen as follows: O128 (*n* = 12, 40.0%) O26 (*n* = 9, 30.0%), O157 (*n* = 6, 20%) and O11 (*n* = 3, 10.0%). The other strains that presented a non-typable O antigen were considered generic *E. coli*, and were not further analysed. Multiplex PCR showed that all 30 strains carried one or more virulence genes (stx1 = 17; stx2 = 16; eae = 21). As detailed in Table 3, the most frequently detected resistances were towards tetracycline (56.6%), followed by ampicillin (50.0%) and ciprofloxacin (33.3%), whereas all strains were susceptible to chloramphenicol (Table 3). The majority of STEC isolates (76.6%) were simultaneously resistant to at least two antibiotics, and nine isolates (30.0%) displayed simultaneous resistance to at least three antibiotics. Specifically, eleven (36.6%) STEC isolates were resistant to ampicillin and tetracycline; four (13.3%) were resistant to ampicillin, tetracycline and enrofloxacin; and two (6.67%) were resistant to ampicillin, tetracycline, and sulphamethoxazole–trimethoprim. Only two (6.67%) *E. coli* O26 strains were positive to the ESBL test.

## 4. Discussion

Enteropathogenic bacteria were detected in 41.3% of the yellow-legged gulls examined in the present survey, including *Salmonella* spp., *Campylobacter* spp., and Shiga toxin-producing *E. coli*. No co-infections were recorded, similarly to a previous survey, focused on two of these enteropathogenic bacteria [10].

Various studies have previously investigated the prevalence of *Campylobacter* spp. in gulls worldwide, but the results are many and heterogeneous. The prevalence of *Campylobacter* spp. reported here (26.7%; 95% CI 21.4–32.8%), as well as the most frequently identified species, differ from other studies [10,13,26]. Migura-Garcia et al. [13] detected 19 *Campylobacter* isolates from 9.3% chicks of yellow-legged gull along the north-eastern Iberian coast, and identified them as *C. jejuni* (65.0%) and *C. lari* (35.0%). Similarly, Broman et al. [26] isolated 250 *Campylobacter* species from 31.8% black-headed gulls (*Chroicocephalus ridibundus*) in southern Sweden, with *C. jejuni* as the most prevalent species (94.0%), followed by *C. lari* (3.2%) and *C. coli* (2.8%). In another survey, Moré et al. [10] isolated thermophilic *Campylobacter* from 12.4% kelp gull chicks (*Larus dominicanus*) in South Africa, with *C. jejuni* as the most frequently identified species, followed by *C. lari*. In the aforementioned studies, *C. jejuni* was predominant, whereas *C. lari* and *C. coli* were less frequently detected. Contrarily, *Campylobacter* strains isolated in our study were identified mainly as *C. coli* (60.0%) and *C. jejuni* (40.0%), whereas *C. lari* was never identified. This contrast might be explained by different gull species and age classes, or by the influence of geographical circumstances, living conditions, feeding habits, and the use of refuse dumps [27].

Concerning antimicrobial resistance, all tested *Campylobacter* strains exhibited susceptibility to chloramphenicol and gentamicin, whereas different rates of resistance were detected towards tetracyclines (16.6–62.5%), fluoroquinolones (29.1–37.5%) and macrolides (11.1–25%). Additionally, 22 strains showed multidrug resistance, defined as resistance to at least three classes of antimicrobial agents [28]. These data are in line with previous surveys that reported resistances towards tetracyclines and fluoroquinolones, although *Campylobacter* resistance to erythromycin, and multidrug resistance, were not reported [10,13]. Indeed, the pattern of resistances as detected in our study is particularly relevant, because macrolides represent the first-line therapy for human *Campylobacter* infections, and tetracycline and fluoroquinolones are considered valid alternatives [29].

*Salmonella* spp. was isolated from only three birds, resulting in a lower prevalence (1.3%) as compared to previous studies [13,27], and identified as *Salmonella enterica arizonae*, although the subspecies *S. enterica enterica* has been more commonly described [10,27]. Curiously, we found a higher occurrence of *Campylobacter* than *Salmonella*, in contrast with the pattern observed in kelp gulls from South Africa, but similar to the greater crested terns examined in the same study [10]. Actually, the prevalence of *Salmonella* in gulls appears variable, and our results are analogous to those reported by Palmgren et al. in black-headed gulls from southern Sweden [30]. As suggested for *Campylobacter*, the differences might be related to distinct locations of colonies and especially to different feeding habits [27]. Gulls are challenged by conditions raised by humans; therefore, these birds might come into contact with contaminated environments, such as surface water polluted by farm effluents, or sewage [31,32]. Another explanation which could be explored is the presence of other potential reservoirs, raised by humans or wild (e.g., chickens, pigeons, corvids, etc.), sharing the same environment inhabited by the gulls [18,31,32,33].

Antimicrobial resistance of *Salmonella* has been previously reported, mainly towards tetracyclines and streptomycin [10,13]. However, all three *Salmonella* strains isolated here exhibited susceptibility to all the tested antimicrobials, excluding sulphonamides, similarly to other studies [10,30]. This is surprising for an urban area such as the city of Naples, because a link has been suggested between the use of urban refuse as a food source and the occurrence of enteric antibiotic-resistant bacteria in gulls [13,27].

Shiga toxin-producing *E. coli* have been largely detected in domestic and wild animals, including gulls [7,14]. Although the prevalence was lower, as compared to other surveys conducted on gulls across Europe [12], the STEC percentage is in line with other studies conducted on wild birds recovered in urban surroundings [34,35]. Unfortunately, it was not possible to establish the O:H serotypes, in order to determine the seropathotypes according to Karmali et al. [36]. However all of our strains carried one or more STEC-associated genes, highlighting the potential role of gulls as a source of STEC to other animals and humans [7,14].

The vast majority (76.6%) of STEC isolated here were simultaneously resistant to at least two antibiotics, and 30.0% of the strains exhibited simultaneous resistance to at least three antibiotics, raising important public health concerns [12,14]. The most commonly detected STEC resistances were towards tetracycline (56.6%) ampicillin (50.0%) and ciproflocaxin (33.3%), data that are in accordance with other studies on gulls; such as one described in Spain, where more than half of the strains exhibited antibiotic resistance [12]. Actually, Stedt et al. [12] highlighted a south-to-north gradient in Europe (valid for *E. coli* from humans, food production animals and gulls), characterized by higher levels of resistance in Mediterranean countries and lower levels of resistance in northern Europe, with few local variations. These results, including ours, reflect the overuse of antibiotics in veterinary and human medicine over the years [12,14], and suggest that gulls might act as vectors but also as victims, acquiring resistant strains that potentially originate from humans or animals, which are often subjected to antibiotic administration [37,38].

*Yersinia* spp. has never been isolated, although this microorganism has been previously isolated from gulls [39]. However, the strains identified in that study were characterized by low virulence and pathogenicity, posing little or no risk to animal and human health [39].

Anthropic activities and gull habits seem to be the main factors involved in the dissemination of resistant bacteria among gulls and other animals, humans, and the environment [12,14]. In the present research, an important role may have been played by the presence of open landfill sites, which are widespread in the study region. Therefore, similarly to other animals inhabiting the marine environment, the occurrence of resistant bacteria in gulls represents an indication of anthropic pressure on the environment and the antibiotic resistome [40,41].

## 5. Conclusions

The present study indicates that yellow-legged gulls might act as reservoirs or carriers of enteropathogenic bacteria, contributing to the epidemiological distribution of such pathogens as well as the potential maintenance and spread of antimicrobial resistance, with potential risks of antibacterial efficacy in human and animal medicine. The reason might be associated to the close contact between gulls and human activities, in particular through parks and shores, which serve as meeting spots for humans, domestic animals, synanthropic birds, and environmental waters. Our findings might be particularly important for other coastal urbanized areas, where gull populations have experienced a significant increase, and where these birds, or their droppings, might come into contact with humans, especially those belonging to the most susceptible age groups (e.g., infants, the elderly, people with immunodeficiencies, etc.), thereby increasing the risk of infection. Further research should be conducted in order to investigate and elucidate the routes through which these bacteria are circulating.

## Figures and Tables

**Table 1:** Prevalence of enteropathogenic bacteria isolated from 225 yellow-legged gulls.

Bacterial Species	Positive Animals (*n*)	Prevalence (95% CI ^a^)	Identification	Strains (*n*)
*Campylobacter* spp.	60	26.7% (21.4–32.8%)	*C. coli*	36/60
			*C. jejuni*	24/60
*Salmonella* spp.	3	1.3% (0.5–3.8%)	*S. arizonae*	3/3
STEC	30	13.3% (9.5–18.3%)	*E. coli* O128	12/30
			*E. coli* O26	9/30
			*E. coli* O157	6/30
			*E. coli* O11	3/30

^a^ CI, Confidence interval.

**Table 2:** Antibiotic resistance of 60 strains of *Campylobacter* spp., isolated from 225 yellow-legged gulls.

Strain	No. of Resistant Strains to Tested Antibiotics (%)								
	AZM	CHL	CIP	CN	DO	E	ENR	NA	TE
*C. coli*	8	0	12	0	6	4	11	10	19
(n.36)	(22.2)		(33.3)		(16.6)	(11.1)	(30.5)	(27.7)	(52.8)
*C. jejuni*	6	0	9	0	5	4	7	9	15
(n.24)	(25.0)		(37.5)		(20.8)	(16.0)	(29.1)	(37.5)	(62.5)

AZM = azythromicin, 15 μg; CHL = chloramphenicol, 30 μg; CIP = ciprofloxacin, 5 μg; CN = gentamicin, 10 μg; DO = doxycycline, 30 μg; E = erythromycin, 15 μg; ENR = enrofloxacin, 5 μg; NA = nalidixic acid, 30 μg; TE = tetracycline, 30 μg.

**Table 3:** Antibiotic resistance of 30 strains of Shigatoxin-producing *E. coli*, isolated from 225 yellow-legged gulls.

No. of Resistant Strains to Tested Antibiotics (%)															
AMP	AMO	AMC	APR	CAZ	CIP	CHL	CS	DO	ENR	CN	NA	S	TE	SXT	ESBL+
15	5	4	4	1	10	0	3	4	5	4	3	1	17	8	2
(50.0)	(16.6)	(13.3)	(13.3)	(3.3)	(33.3)		(10.0)	(13.3)	(16.6)	(13.3)	(10.0)	(3.3)	(56.6)	(26.6)	(6.6)

AMP = ampicillin, 10 μg; AMO = amoxicillin, 30 μg; AMC = amoxicillin–clavulanate, 20  +  10 μg; APR = apramycin, 40 μg; CAZ = ceftazidime, 30 μg; CIP = ciprofloxacin, 5 μg; CHL = chloramphenicol, 30 μg; CS = colistin sulphate, 10 μg; DO = doxycycline, 30 μg; ENR = enrofloxacin, 5 μg; CN = gentamicin, 10 μg; NA = nalidixic acid, 30 μg; S = streptomycin, 10 μg; TE = tetracycline, 30 μg; SXT = sulphamethoxazole–trimethoprim, 1.25  +  23.75 μg; ESBL+ = Extended Spectrum Beta-Lactamase production.

## Data Availability

The dataset used and/or analysed during the current study are available from the corresponding author on reasonable request.
